# Receptor Density-Dependent Motility of Influenza Virus Particles on Surface Gradients

**DOI:** 10.1021/acsami.3c05299

**Published:** 2023-05-11

**Authors:** P. H. Erik Hamming, Nico J. Overeem, Kevin Diestelhorst, Tren Fiers, Malte Tieke, Gaël M. Vos, Geert-Jan P. H. Boons, Erhard van der Vries, Stephan Block, Jurriaan Huskens

**Affiliations:** † Molecular Nanofabrication Group, MESA+ Institute, Faculty of Science and Technology, University of Twente, P.O. Box 217, 7500 AE Enschede, The Netherlands; ‡ Institute of Chemistry and Biochemistry, Freie Universität Berlin, 14195 Berlin, Germany; § Division of Virology, Department of Infectious Diseases and Immunology, Faculty of Veterinary Medicine, Utrecht University, 3584 CL Utrecht, The Netherlands; ∥ Department of Chemical Biology & Drug Discovery, Utrecht Institute for Pharmaceutical Sciences, Bijvoet Center for Biomolecular Research, Utrecht University, 3584 CG Utrecht, The Netherlands; ⊥ Complex Carbohydrate Research Center, University of Georgia, 315 Riverbend Road, Athens, Georgia 30602, United States; # Department of Chemistry, University of Georgia, Athens, Georgia 30602, United States; ∇ Royal GD, Arnsbergstraat 7, 7418 EZ Deventer, The Netherlands; ○ Department of Clinical Chemistry and Haematology, University Medical Center Utrecht, Utrecht University, 3584 CX Utrecht, The Netherlands

**Keywords:** motility, influenza, surface gradients, multivalency, receptor density, surface diffusion

## Abstract

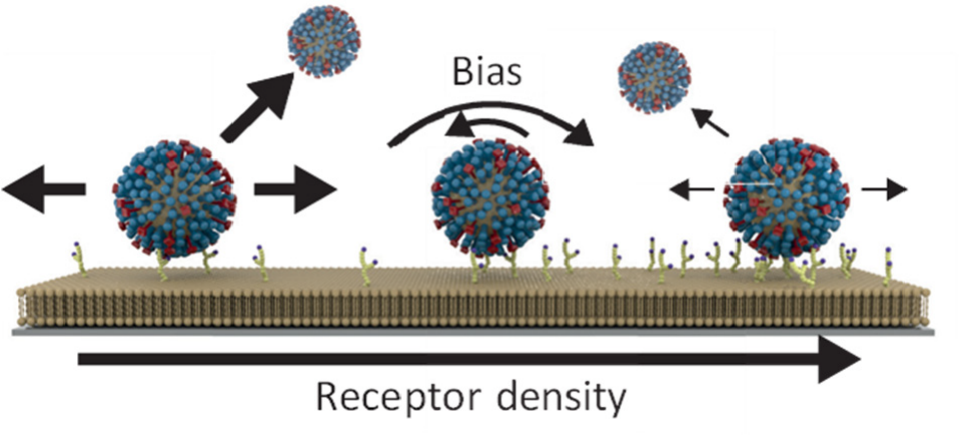

Influenza viruses can move across the surface of host cells while interacting with their glycocalyx. This motility may assist in finding or forming locations for cell entry and thereby promote cellular uptake. Because the binding to and cleavage of cell surface receptors forms the driving force for the process, the surface-bound motility of influenza is expected to be dependent on the receptor density. Surface gradients with gradually varying receptor densities are thus a valuable tool to study binding and motility processes of influenza and can function as a mimic for local receptor density variations at the glycocalyx that may steer the directionality of a virus particle in finding the proper site of uptake. We have tracked individual influenza virus particles moving over surfaces with receptor density gradients. We analyzed the extracted virus tracks first at a general level to verify neuraminidase activity and subsequently with increasing detail to quantify the receptor density-dependent behavior on the level of individual virus particles. While a directional bias was not observed, most likely due to limitations of the steepness of the surface gradient, the surface mobility and the probability of sticking were found to be significantly dependent on receptor density. A combination of high surface mobility and high dissociation probability of influenza was observed at low receptor densities, while the opposite occurred at higher receptor densities. These properties result in an effective mechanism for finding high-receptor density patches, which are believed to be a key feature of potential locations for cell entry.

## Introduction

Influenza has a large and worldwide impact, both seasonally and through occasionally occurring pandemics. While the complete workings of the influenza virus are complicated, influenza as a particle can be studied and understood from the perspective of supramolecular and physical chemistry. Influenza is a virus that has high numbers of mainly two proteins on its surface, hemagglutinin (HA) and neuraminidase (NA), which are densely but typically unevenly distributed.^1−3^ On the level of individual proteins, the role of HA is its binding to cell receptors (primarily sialic acid-terminated glycans, favoring galactose and *N*-acetyl glucosamine as second and third moieties, attached to membrane protein or lipid) while NA binds and cleaves some of these receptors (removal of the terminal sialic acid). Sakai et al. reported on the role of NA in the surface motility of influenza.^4,5^ De Haan et al. found that viruses cleave receptors in a large area around them and proposed that viruses roll over cell surfaces.^6^ Vahey and Fletcher found evidence of directional motion by influenza,^1^ while Müller et al. showed that the mobility of influenza relies on a delicate balance between binding and cleaving of receptors.^7^ The surface motility of influenza may assist in finding or forming clathrin-coated pits. Clathrin-mediated endocytosis is influenza’s main method of cellular uptake.^8−11^

In previous work, we established that the thermodynamic, multivalent binding of influenza viruses can be quantitatively understood by regarding it as a ligand-decorated particle binding to a receptor-decorated surface assuming independent interactions.^12,13^ In these studies, we always used an NA inhibitor; hence, we could only view the HA-involved noncovalent binding to sialic acid receptors. The virus binding shows rich multivalent binding behavior, which is explained by the theory of superselectivity.^14,15^

Virus motility (the term “motility” is used here for all forms of mobility where an active process is involved, here primarily the enzymatic activity by NA), however, involves catalytic cleavage of sialic acid end groups from these receptors by NA; without NA, virus motion is commonly only diffusive. Therefore, we recently reviewed the observed motility of influenza from the perspective of molecular walkers, which are molecular systems in which similar reactions occur between surface receptor groups and binders that attach to them from solution.^16^ The comparison between influenza and molecular walkers results in a set of predictions of influenza behavior that are dependent on receptor density. When the receptor density is inhomogeneous, binding an additional receptor by influenza is statistically more likely on the high-density side; thus, we expect the movement of influenza to have a bias toward higher densities.^17^ Furthermore, while surface-bound, we expect influenza to diffuse faster at low receptor densities, but with shorter residence times, than at high receptor densities. The combination of these behaviors constitutes an efficient method of finding a high-receptor density area.^18^ Coincidentally, de novo formation of clathrin-coated pits around influenza requires at least six clathrin proteins, indicating a role of receptor density also in a biological context.^8^

Surface gradients with gradually varying receptor densities could thus be a valuable tool to study binding and motility processes of influenza. Influenza binding to a receptor density gradient should result in both bias and density-dependent behavior. Both molecules and nanoparticles have been shown to exhibit (passive) motion under the influence of a surface gradient.^19−22^ Surfaces with a receptor density gradient have been shown to be powerful tools to study thermodynamic density-dependent behavior, such as superselective binding.^12,13^ In a slightly different setup, the same gradients can be used to assess kinetic density-dependent behavior of influenza-surface binding.

In this study, we track influenza virus particles while moving over receptor density gradients with the aim to study how the surface gradient affects the dynamic properties of the virus-surface binding. First, we discuss the state of the art of studying influenza virus mobility and what we expect regarding the effects of receptor density on virus mobility. Then, we describe the formation and analysis of the receptor density gradients used here. Virus particles are then imaged on these gradients by means of fluorescence microscopy, and the virus tracks are extracted and discussed at a general level, verifying their validity and activity. Hereafter, we assess the contribution of NA activity, and we quantify and discuss the populations of mobile and immobile viruses. The effects of receptor density on (i) a possible bias of virus motion, (ii) diffusion rate, and (iii) dissociation rate are analyzed and evaluated.

## Results and Discussion

### Density-Dependent Surface Motility of Influenza

The interaction between an influenza particle and a receptor-decorated surface is multivalent. The overall binding is determined by both the number of interactions as well as the strength of each individual interaction. The individual interaction strength is mostly dependent on the strain of influenza and the type of receptor (either human-type or avian-type). In this project, the Influenza A Puerto Rico/8/1934 strain has been used; this strain binds to both types of receptors with mM affinity.^23−25^ In addition to influenza strain and receptor type, receptor *density* is an important factor in virus binding. The interaction area between virus and surface is limited by the distance that receptors and proteins can bridge, and thus, it is the receptor density that determines the maximum number of interactions. As the contribution by individual interactions is low, the total interaction strength—and more generally the surface binding and dynamics—of influenza viruses is strongly dependent on the number of interactions.

Influenza particles can bind receptors either through its HA or NA proteins. Whereas HA binds reversibly to receptors, NA can irreversibly cleave off the terminal sialic acid residue of the receptor it binds to, thereby nearly eliminating the affinity for it. When the NA activity is suppressed, only slow and diffusive virus mobility is observed; the surface-bound motility of influenza particles thus requires the receptor-destroying action of NA.^4,6^ The balance of HA and NA determines virus binding, but as NA cleaves receptors, the local receptor gradient resulting from this cleavage process is assumed to function as a driving force that propels the virus particle away from the cleaved site.

This study does not distinguish between the contributions of either protein, it considers the virus as a whole, mobile entity. The arguments why density-dependent motility is expected, laid out below, therefore apply to influenza particles (with geometrically separated binding and cleaving) and also to particles that combine binding and cleaving within a single protein (such as some influenza strains, which bind primarily through NA^26,27^) and even to particles that bind weakly and reversibly, without any cleaving action.

**Figure 1:**
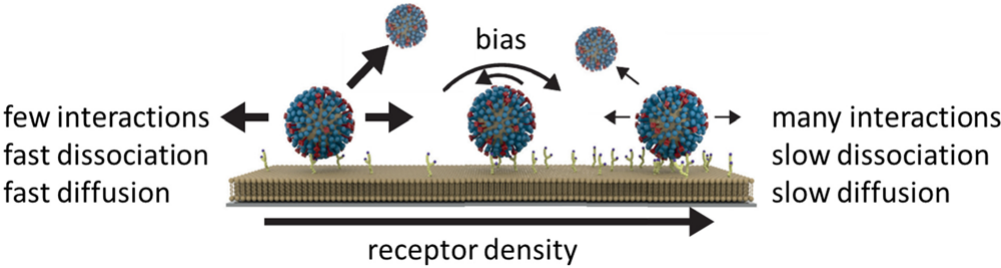
Density-dependent motility of the influenza virus. Using a receptor density gradient allows for the simultaneous observation of influenza particles at a range of (local) receptor densities. If the local receptor density around an influenza virus is nonhomogeneous, a bias toward higher receptor densities is expected. At low receptor densities, the number of interactions that can be formed will be low. A low number of interactions should result in faster diffusion at the expense of higher dissociation. At high receptor densities, the number of interactions that can be formed will be high. A high number of interactions inhibits diffusion and also decreases dissociation.

Receptor density gradients provide a powerful surface architecture to study density-dependent surface motility of particles, as shown in Figure 1. A bias toward higher receptor densities is expected when an influenza virus particle faces a gradient in receptor density. The explanation is found in the argument for superdiffusivity as presented by Stefanovic et al.^28^ When bound at the surface, individual ligand–receptor pairs may break or (re)form. When a ligand–receptor pair on the outer edge of the contact area is broken, the virus gains some freedom of movement to probe the area around its contact area and form a new ligand–receptor pair. The probability of breaking a ligand–receptor pair is uniform, but the probability of (re)binding a receptor depends on the number of free receptors within range of a ligand and thus on the receptor density. As the receptor density gradient is continuous, the same logic applies at every rolling step of influenza.

The number of interactions between an influenza particle and the surface determines the diffusion speed and dissociation probability. Dissociation of a particle from the surface requires all ligand–receptor pairs to be unbound at the same time. The overall dissociation rate constant for such a particle bound through multivalent, independent interactions, is determined by the intrinsic dissociation rate constant of a single interaction and the fraction of the bound particle that is connected through only a single bond.^29^ For increasing numbers of bonds between a particle and the surface, the latter fraction decays exponentially with the average number of ligand–receptor pairs. As the number of interactions increases, the average distance between interactions decreases. The implication of this is that the step size decreases. A step of influenza in this sense is a complete cycle of the unbinding of a ligand–receptor pair on the edge of the contact area, influenza gaining freedom of movement and probing the surrounding area, and the forming of a new ligand–receptor interaction. The maximum distance covered by any one step is therefore determined by the distance between interaction pairs.

In the simplest case, the number of interactions is only dependent on the number of receptors available. The density of ligands on a virus particle generally exceeds the maximal receptor density of the gradient, while each binding site is typically within the range of (at most) just a single receptor.^12^ This essentially makes the receptor density the sole determinant for the number of interactions. Influenza particles bound with few interactions are therefore more likely to be found—but not exclusively—at the low receptor density areas.

### Surface Receptor Density Gradients

A receptor density gradient allows us to study each density-dependent behavior on a variety of densities at the same time. In previous work,^12,13^ receptor density gradients have been used to study the thermodynamics of the binding of influenza to receptor surfaces. The coverage of influenza ranged from near-zero to full coverage over a 10-fold increase in receptor density at the gradient. Such a gradient is thus broad enough to let influenza exhibit the whole range of thermodynamic binding behaviors, from no to very strong binding; to what extent it is sufficiently broad to see the full range of dynamic behaviors, as presented in Figure 1, will be assessed here.

**Figure 2:**
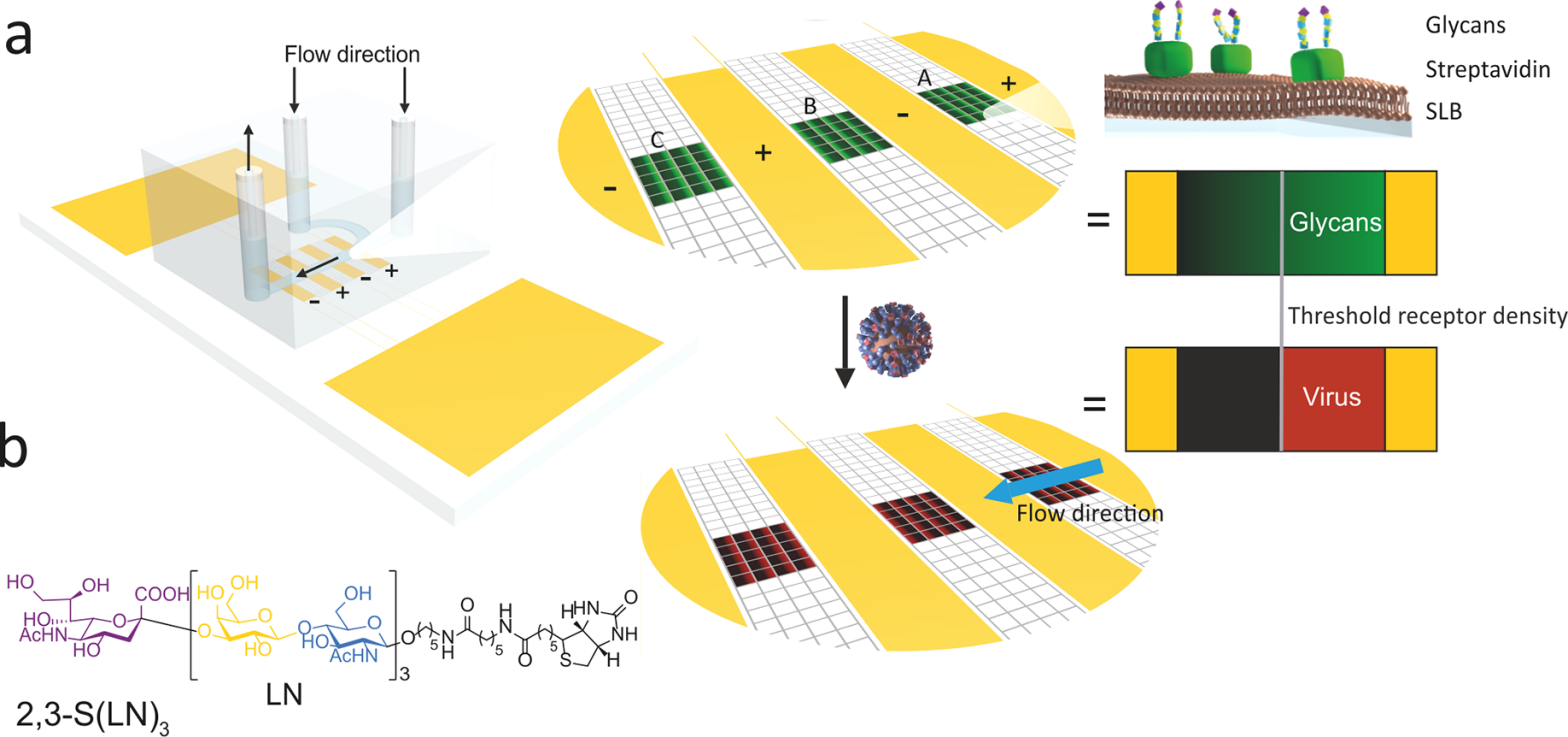
Method for visualization of superselective binding of IAV. (a) Method to form surface receptor density gradients. In SLBs between electrodes inside a microchannel (positions A–C), electrophoretic gradients of biotinylated lipids are formed. Fluorescently labeled SAv and 2,3-S(LN)_3_ are bound onto the biotinylated lipids to form a receptor gradient. Fluorescently labeled influenza virus is used. (b) Structure of the biotinylated glycan receptor 2,3-S(LN)_3_. In the dummy receptor (LN)_2_, which is used as a negative control for 2,3-S(LN)_3_, the sialic acid group (purple) and one LN repeat are omitted. Reproduced from ref (13). Copyright American Chemical Society.

The method of gradient formation has been described elsewhere.^12,13,30−33^Figure 2 shows the microfluidic chip in which the receptor density gradients are formed. In short, gradients are formed by applying an electric field to a heated, biotinylated SLB consisting of 1-myristoyl-2-palmitoyl-*sn*-glycero-3-phosphocholine (MPPC) with a small fraction (0.5%) of 1,2-dioleoyl-*sn*-glycero-3-phosphoethanolamine-*N*-(biotinyl) (DOPE-biotin). An SLB provides good antifouling properties necessary to avoid nonspecific interactions with virus particles, while the minor biotin–lipid fraction controls the average glycan density at the final gradient surface. The electric field induces directional motion of the charged biotin–lipid and the resulting lipid redistribution provides the desired gradient, here with local biotin % ranging from about 0.2 to 2%. Upon cooling (in the presence of the electric field), the SLB solidifies into a gel-state SLB, and the gradient is frozen in. The biotin in the SLB provides a means to further functionalize it using streptavidin (SAv) and biotinylated receptors.

After preparing gradients functionalized with the (avian) 2,3-S(LN)_3_ receptor, a solution of influenza virus (Puerto Rico/8/1934, Mt. Sinai) particles with an R18 fluorescent label was passed over the surface at a flow rate of 5 μL/min. Fluorescence micrographs were obtained using a 40× oil immersion lens and a high-quantum yield camera. Under these conditions, individual virus particles could be imaged over multiple frames without significant bleaching. Image analysis of these gradients has been described before^13^ and results in receptor densities at each pixel (see the Supporting Information (SI), Figure S1).

Depending on camera position, the flow can either be with or against the gradient. The (thermodynamic) binding profiles of influenza viruses binding on a gradient with or against the flow are identical.^13^ Flow rate does influence virus binding independent of gradient direction, with the flow rates as used here acting as a shear force that promotes virus dissociation. As an indication, the drag force on a 112 nm sphere at the bottom surface of the channel at a flow rate of 5 μL/min was found to be 42.5 fN by finite element simulations or 130 fN using an analytical model for a tethered particle.^13^ These forces are smaller than the rupture force of a single HA-SA bond by AFM^34^ but skew the energy landscape and potentially hinder rebinding. On the scale of individual viruses, the flow direction may thus bias the movement and as such is indicated on relevant graphs shown below.

### General Track Analysis

**Figure 3:**
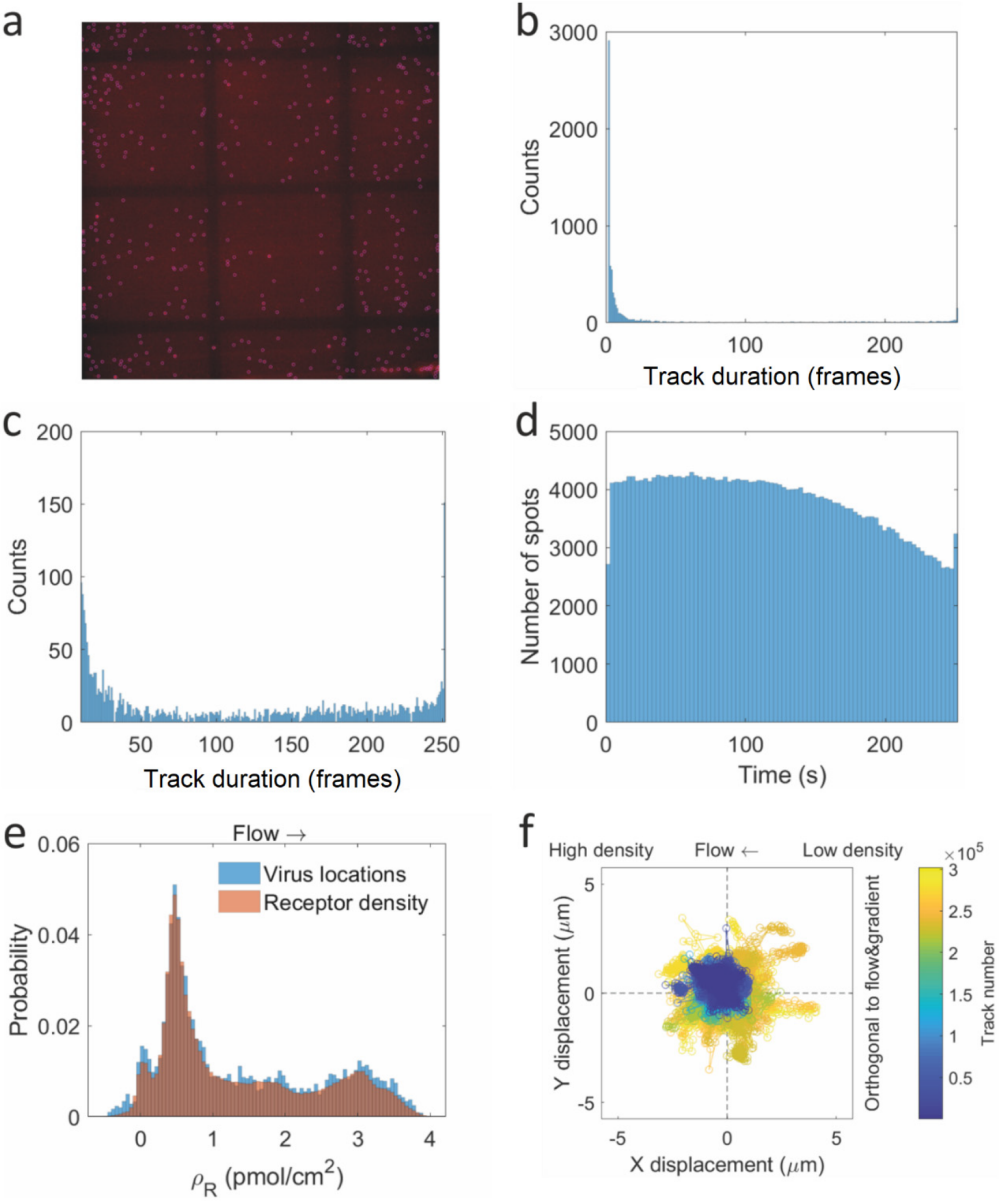
(a) Fluorescence micrograph of fluorescent dye-labeled influenza viruses, in which detected virus particles are enhanced digitally (original in Figure S2). Using an automated detection algorithm, even the virus spots with low signal-to-noise ratio can be tracked. (b) Histogram of track duration for all tracks, and (c) histogram of track duration for tracks of at least 10 frames. (d) Counts of the number of virus/spots per frame, which shows decreasing coverage over time. (e) Histogram of the receptor density at which a virus spot is detected (blue) and histogram of receptor densities *ρ*_R,i_ (orange), showing similar virus coverage regardless of receptor density. (f) Superimposed tracks centered on (0,0) showing the absolute scale of virus movement.

We begin the analysis of virus tracks on a very general level to assess the length and time scales of virus movement, as well as to verify that the movement is NA-related. Figure 3a shows a fluorescence micrograph of the SLB surface, with the detected virus locations indicated by circles. Figure 3b shows a histogram of the durations of all tracks, while Figure 3c shows the same histogram, but for tracks with at least 10 frames. The track duration (in number of frames) is synonymous here to track lifetime, since a constant frame rate is used. Taking only the longer tracks into account significantly decreases the computational load while focusing the subsequent analysis on the longer-term behavior of individual influenza viruses. In both cases, the histograms decrease sharply with increasing track duration at first but level off and have a single upward peak at the end.

The probability for a virus to dissociate increases with time, congruent with a virus that actively cleaves its own receptors using NA faster than it ‘replenishes’ its receptors by moving to a fresh area. Virus dissociation requires all virus–surface interactions to be dissociated at the same time. If there is no cleaving action by NA, the number of virus–surface interactions is expected to be roughly constant, leading to a roughly constant overall virus dissociation rate coefficient. If, however, NA is active, the number of virus–surface interactions is expected to decrease over time, resulting in a dissociation rate coefficient that is increasing with time. When plotting the data of the histogram shown in Figure 3c on a semilog axis (data not shown), the dissociation rate coefficient appears to be increasing with time. This is a first indication that we are looking at NA-dependent behavior.

The leveling off of the counts as a function of track duration (Figure 3b,c) indicates that not all viruses dissociate. One possibility is that these viruses move to fresh areas faster than their NA can cleave receptors. Movement would reduce dissociation rates but is unlikely to stop dissociation of influenza entirely. As influenza viruses are patchy in their spatial distribution of HA and NA (and a virus sample is likely to have particles with a distribution of HA/NA ratios), it is more likely that these tracks relate to viruses that are bound with a face showing only HA and no NA. Lastly, they could be fixed artifacts that are mistakenly labeled as virus, but this level of general analysis does not identify that. The final peak is an artifact due to the fact that tracking was stopped after 250 frames.

The effect of NA is also visible in the virus density. Figure 3d shows the number of spots in each frame over time. As opposed to Figure 3b,c, this number is not related to the duration of tracks but instead gives the average density of viruses. Over time, there is a steady decrease in the number of spots per frame, excluding the very last frame.

The decreasing number of spots over time is not an effect of photobleaching. Spots of viruses that have been tracked for 200 frames or more, on average, decreased in intensity by 0.33% over the total duration of tracking. The effect of photobleaching is thus not strong enough to explain the decreasing number of virus spots. Instead, the decreasing number of spots over time in Figure 3d indicates that viruses are dissociating faster than binding. One explanation would be that viruses are moving over the surface and cleaving receptors, reducing the binding rate of new viruses. This would match what Guo et al. observed.^6^ Another explanation is that their probability to dissociate increases with time as they cleave their surrounding receptors. This matches the behavior from Figure 3b,c. In case of the first explanation, rolling viruses, we expect the effect to be much stronger at lower receptor densities as the residual receptor density after cleaving at high receptor densities may be enough to still facilitate virus binding.

Figure 3e shows the probability of finding a particular receptor density value (*ρ*_R,i_) within the sample (i.e., taking the entire image into account), in comparison to the corresponding distribution of *ρ*_R,i_, if only virus-engaged pixels are taking into account. Differences between these histograms would indicate that virus binding/coverage is receptor density dependent. Instead, Figure 3e shows no clear preference in virus binding at a given receptor density or any thermodynamic behavior whatsoever. This matches previous work, where it took at least several hours to see a binding profile.^13^Figure 3d,e thus further indicates that there is NA-dependent behavior but on the scale of single viruses/tracks and not on a substrate-wide length scale.

The length scales of tracks (i.e., their spatial extent) can be visualized by plotting them superimposed with all starting points moved to (0,0), as shown in Figure 3f. The *x* and *y* axes are in micrometers, thus giving the absolute distance moved by each virus after binding to the surface. This gives a measure for the maximum area probed and thus for the maximum area at which a virus cleaves receptors and thereby prevents other viruses from binding. The direction of the receptor density gradient (high/low) is indicated in Figure 3f, as well as the direction (arrow) of the flow. The length scales of the tracks in Figure 3f clearly supersede virus diameter. The movement thus indicates true motion where all original ligand–receptor pairs have to dissociate and new ones form, as opposed to motion over nanometer length scales, which could be explained by a virus wiggling in place. The gradients are almost perfectly left–right aligned. Virus tracks having a bias toward higher receptor density should result in an elliptical shape wider in the horizontal than vertical direction and with its center toward the high-receptor density side. For now, we conclude that any possible bias in motion is of limited effect, as the shape of Figure 3f is roughly circular and centered around (0,0). In the discussion below, we analyze the possible directional bias in more detail.

### Virus Mobility

When influenza viruses are introduced to a surface, there is typically a fraction of immobile viruses.^7^ Based on the histogram of track durations in Figure 3c, a fraction of immobile viruses is also expected in our experiments. It would be informative to quantify the fraction of viruses that are immobile. To this end, we performed an analysis of the mean squared displacement (MSD) of the IAV trajectories. The MSD of an individual IAV trajectory is calculated using
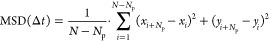
1with *x*_*i*_ and *y*_*i*_ being the *x* and *y* coordinates of frame *i*, *N* the number of frames of the track, and *N*_p_ the frame displacement used to calculate the MSD value. The property *N*_p_ can be translated into the lag time Δ*t* by multiplication with the time period Δ*t*_0_ between two consecutive frames: Δ*t* = *N*_p_·Δ*t*_0_. In order to avoid misinterpretations that arise at too large *N*_p_ values,^35^ the MSD(Δ*t*) curve was calculated to a maximum frame displacement of 10% of the track duration: *N*_p_ ≤ 0.1·*N*.

**Figure 4:**
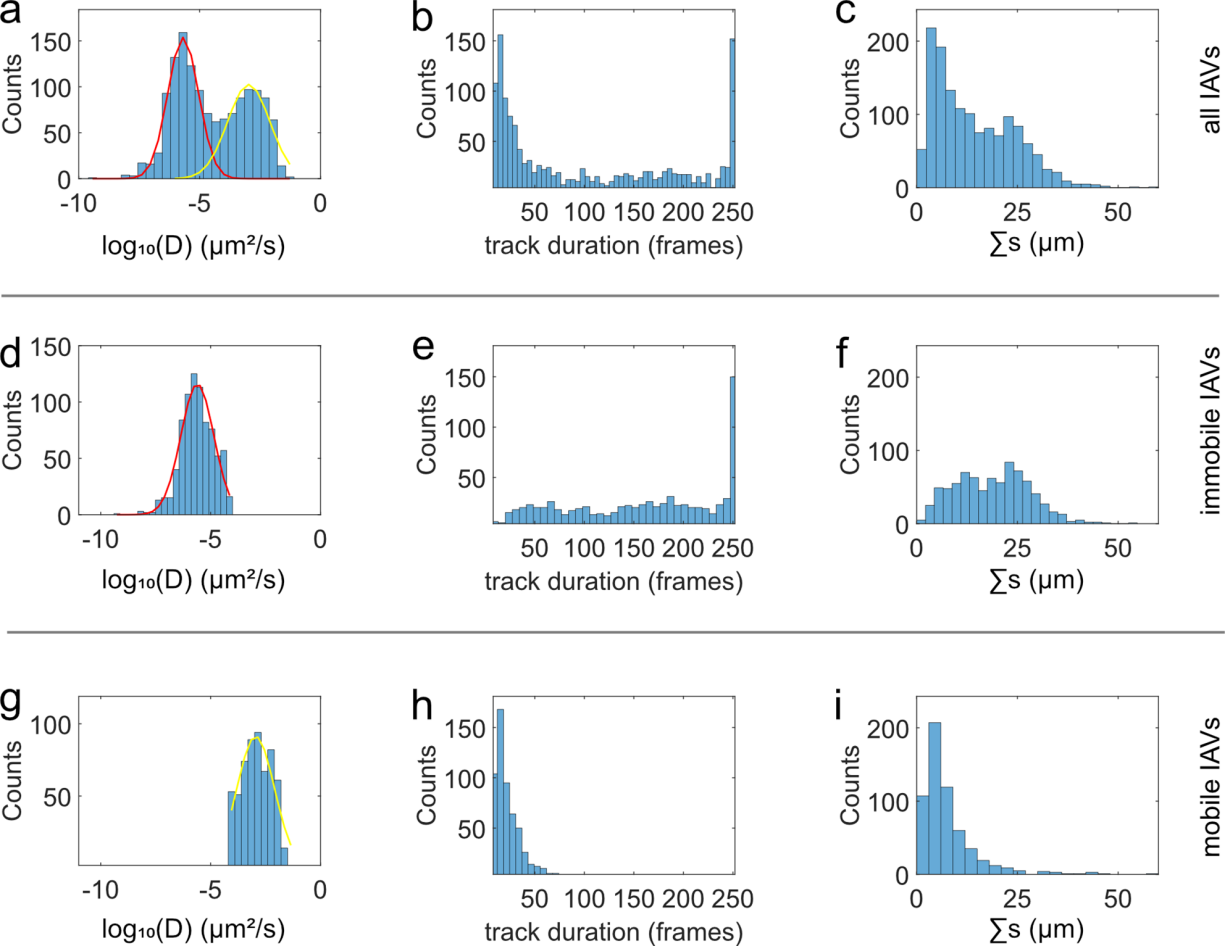
Analyzing the fractions of mobile and immobile viruses within the population. (a) Histogram (blue) of the observed diffusion coefficients with a double Gaussian function fitted (red/yellow). Two populations are observed, which correspond to immobile (red) and mobile IAVs (yellow population), respectively. (b and c) Histogram of the track durations and of integrated distance traveled (see eq 2) of all tracks. Both distributions also show indications for the presence of two populations that may correspond to the population of immobile and mobile IAVs observed in (a). Indeed, restricting the calculation of these histograms to immobile (d–f) and mobile IAVs (g–i) reveals that mobile IAVs tend to dissociate within 50 frames (h), whereas long residence times (>50 frames) are predominantly observed (e) for immobile IAVs.

The MSD analysis revealed that the majority (>90%) of the IAV trajectories showed a linear dependence on lag time Δ*t*. The slope of the MSD(Δ*t*) curves therefore allows to extract the diffusion coefficients of the tracked IAVs, the histogram of which shows two distinct populations centered at approximately 10^–6^ μm^2^/s and 10^–3^ μm^2^/s (Figure 4a). These populations are attributed to mobile (∼10^–3^ μm^2^/s) and immobile IAVs (∼10^–6^ μm^2^/s), whereas the nonzero value of the latter is caused by the finite accuracy in localizing the position of the IAVs (localization error, approximately 50 nm, Figure S3).

In addition to the diffusion coefficients, Figure 4 also shows the track duration distribution of the obtained IAV trajectories (Figure 4b) as well as the distribution of the integrated distance, Σ*s*, traveled by the IAVs (Figure 4c), which is defined by
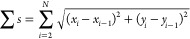
2

This property measures only the *apparent* integrated distance traveled by a particular IAV; it nears the real integrated distance traveled in the limit of time between frames Δ*t* = 0. For our purpose, the apparent integrated distance traveled suffices.

The population of mobile IAVs (yellow in Figure 4a; 42% of all tracks) predominantly shows short track durations (Figure 4b,h), and the histogram of track duration matches the sharp decline that was observed in Figure 3c but lacks the upward trend at the end. This suggests that this fraction indeed represents a—probably not homogeneous—population of mobile viruses. It is this population that exhibits the full range of receptor density-dependent behavior shown below. In contrast, the population of immobile IAVs (red in Figure 4a; the remaining 58%) shows a track duration histogram (Figure 4e), suggesting that the average track duration is in excess of the experimental time frame, with the viruses of this population responsible for the upward trend noted in Figure 3c. As the red population is the immobile population, the integrated distance traveled must be solely due to fluctuations in intensity that are mistakenly tracked as motion, which is in line with the localization error described above (Figure S3).

Surprisingly, both mobile and immobile viruses occur at all glycan densities and are distributed rather independently of glycan density (Figure S4). This indicates that the fractions of mobile and immobile viruses are predominantly determined by the heterogeneity of the virus sample rather than by surface properties. As mentioned above, hemagglutinin (HA) and neuraminidase (NA) are densely but typically asymmetrically distributed.^1−3^ Using fluorescent labeling, Vahey et al.^36^ found densities of ∼22,800 HA and ∼2090 NA per μm^2^, corresponding to 340 HA trimers and 24 NA tetramers for a spherical virus of 120 nm. Using cryo-TEM, Harris et al.^2^ counted the number of spikes on two well-resolved X-31 virions to be 301 HA trimers plus 50 NA tetramers and 290 HA plus 38 NA, respectively. Vahey and Fletcher^1^ defined the polarity of filamentous viruses as the separation between the center of mass of HA and NA divided by the length of the virus and found (for A/WSN/1933 with M1 from A/Udorn/1972) values of 0.124 ± 0.012. These studies confirm heterogeneity of virus samples, in size, shape, and ratios and spatial distributions of HA and NA. These heterogeneities may result in highly different binding and mobility behavior, as observed here.

Assuming each track belongs to a unique virus particle, the fractions of the tracks in each population reflect the fractions of mobile and immobile viruses. In Figure 4, 42% of the tracks are classified as mobile viruses, compared to ∼45% reported for a different influenza strain moving over an SLB.^7^ This fraction may be even slightly smaller since a single, mobile virus particle may generate multiple short tracks. The larger fraction (58%) is given by immobile IAVs; the exact reasons why an immobile fraction is often observed, are unknown. This fraction may either be poorly formed viruses or simply viruses bound with a face that only expresses HA. HA and NA are densely packed on the surface, in an approximately 6:1 ratio.^2^ Though patches and even complete separation of HA and NA into domains have been reported,^1−3^ this is not known to be the case for the strain used in our study. Given the number of proteins, it is unlikely that all of the immobile viruses are bound with a face not expressing any NA. As a rough estimate, using a 6:1 HA/NA ratio, a 112 nm virus particle, and a 4% contact area with 3.8 pmol/cm^2^ site density, we can estimate about 11.5 proteins or 1.65 NA in the contact area, which leads, using a Poisson distribution, to a probability of 19% to find zero NA in the contact area. This value is significantly lower than the here observed immobile fraction, although the estimated probability could be higher when strong phase separation of HA and NA is assumed. An explanation may lie in that a lab strain of virus was used in these experiments, as such a large fraction of viruses not contributing to infection in a biological setting seems to be a very ineffective use of resources. On the other hand, the average time a mobile virus remains at a surface is much shorter. The mobile viruses may dissociate and bind elsewhere; the effectivity of this mechanism may be high enough to overcome the downside of having immobile viruses. We thus proceed with the analysis, taking only the *mobile* fraction of viruses into account.

### Bias of Virus Mobility toward Higher Receptor Densities

Reducing the dimensionality of the data makes quantitative analysis computationally easier. As the local receptor densities in each pixel, ρ(*x*,*y*), are known, (*x*,*y*,*t*) coordinates can be converted into (ρ,*t*) coordinates by bilinear interpolation. Instead of a 2D plot in (*x*,*y*) such as in Figure 3f, the tracks can be shown as receptor density over time (Figure S5). As the virus cleaves receptors, the local receptor density will drop. This effect is not captured by our experimental method of determining the receptor density and thus the reported receptor density always refers to the *initial* receptor density at that position on the surface.

The tracks contain high-frequency fluctuations as well as longer time scale trends. The fluctuations may be caused by noise in the data, which interferes with the subpixel localization, but it may also result from viruses wiggling around their binding area due to Brownian motion. By filtering it using a 10 s moving mean, the longer time scale features are brought out (Figure S5b). The lines are close to linear and without an obvious slope up or down. This indicates there is no clear transient behavior and the tracks can be further simplified without losing too much information. Instead of analyzing every step or every spot, tracks can be analyzed as a whole entity and simplified to their average receptor density or total change in receptor density. Doing so, the analysis reduces the dimensions of the data from three (*x*,*y*,*t*) to two (*ρ*_R_,*t*) to one (Δ*ρ*_R,track_ or < ρ_R,track_>), and unlocks additional analysis methods through which biases and trends can be identified more clearly.

**Figure 5:**
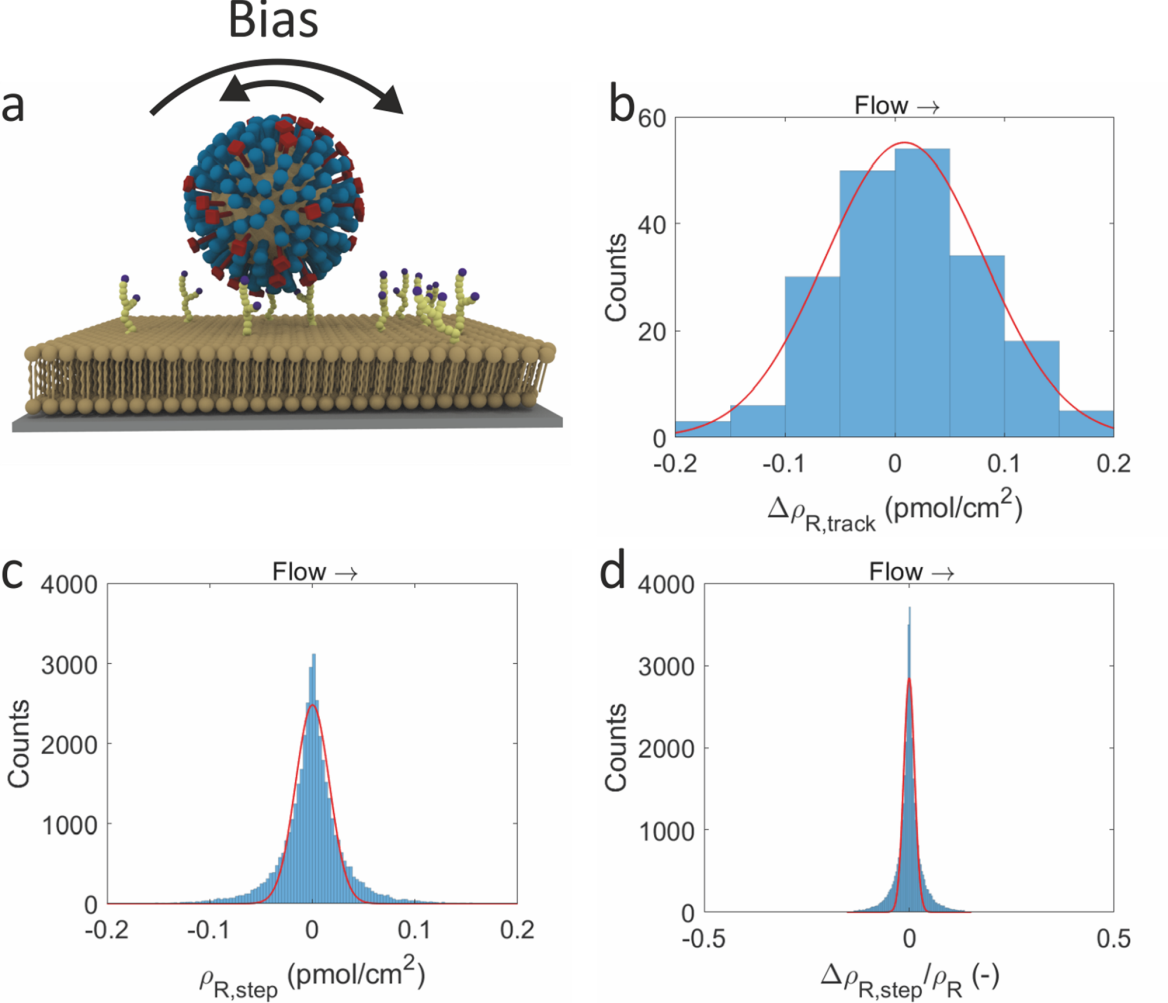
Receptor density bias. (a) Bias toward higher receptor densities is expected as the probability of binding a receptor is higher on the side with more receptors. (b) Histogram of the total receptor density difference over a track (blue bars) with indicated Gaussian fit (red line). Fit centered at 0.0012 ± 0.0034 pmol/cm^2^ (95% ci). (c) Histogram of the receptor density difference for each step (blue bars) with indicated Gaussian fit (red line). Fit centered at 3.4·10^–4^ ± 6.8·10^–4^ pmol/cm^2^ (95% ci). (d) Histogram of the relative receptor density difference for each step (blue bars) with indicated Gaussian fit (red line). Fit centered at 6.6·10^–4^ ± 5.9·10^–4^ pmol/cm^2^ (95% ci).

Above it was explained why a bias of virus motility toward higher receptor density is expected (Figure 5a). The relative probability for any one step to either move up or down the gradient should be proportional to the receptor density on that side of a virus particle. While a small bias is expected each step, viewing tracks as a whole may show the effect more clearly. Figure 5b shows a histogram of the total receptor density change over the duration of each of the tracks. A Gaussian is fitted to the histogram, centered on 0.0012 pmol/cm^2^ and with a 95% confidence interval of 0.0034 pmol/cm^2^. Figure 5c shows a histogram of the receptor density change per step, and Figure 5d shows the histogram of the relative receptor density change per step.

The histogram in Figure 5b shows a bias neither to the lower nor to the higher receptor density. This is further illustrated by looking at the step size in Figure 5c. Over the course of a full track of tens of frames (Figure 3b,c), the total displacement is of a similar scale as each individual step. The fitted Gaussian in Figure 5c also shows this since the 95% confidence interval of the bias is much larger than the fitted bias. Interestingly, the histogram in Figure 5c shows clear kurtosis, which means that the distribution of step sizes is not a normal distribution, which in turn means that not all steps are equivalent. The first assumption would be that the receptor density change per step is higher where the absolute receptor densities are higher. The histogram in Figure 5d is corrected for this effect and shows an even higher kurtosis. The next explanation is then that there are faster and slower steps, for instance, related to local, uncleaved receptor density. The lack of kurtosis in Figure 5b is explained by each track either going through multiple ‘phases’ or that the viruses at faster tracks dissociate sooner. In any case, the receptor difference over the complete track does not show any bias and as such does not constitute a search mechanism toward higher receptor densities on a macroscale, as shown schematically in Figure 1.

Overall, the data above do not indicate the presence of a bias, and also, the flow direction (indicated in Figure 5b–d) does not appear to matter. The reason a bias toward higher receptor densities is not observed may be that the surface receptor gradient is not steep enough. Assuming the gradient is a perfect exponential with a 10-fold increase in density over the 100 μm of the gradient,^31^ the difference in receptor density to the left and right of an influenza virus particle (100 nm in diameter) is 0.23%. This would, at maximum, result in a 0.23% higher probability to move up the gradient instead of down. In reality, the contact area that the virus senses is much smaller than 100 nm, and the gradient is not perfectly exponential, which may—on a molecular scale—be flat locally. Moreover, in future work, theoretical estimations of the driving force may be used to design the necessary steepness of such a gradient to observe directional motion.^37^

### Receptor Density-Dependent Diffusion and Dissociation Rates

**Figure 6:**
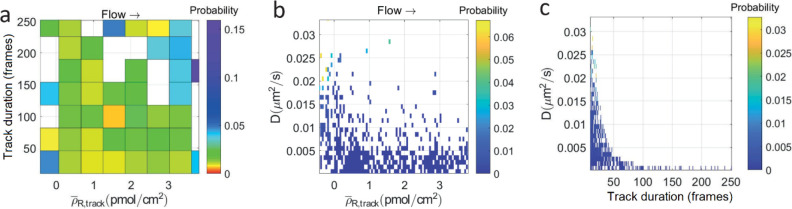
Density-dependent diffusion and dissociation. (a) 2D histogram of the track duration (in frames) versus the receptor density. (b) 2D histogram of the virus diffusion coefficient versus the receptor density. The bins in *x*-direction are identical to, and normalized by, the receptor density histogram in Figure 3e. The bins in *y*-direction are identical to, and normalized by, the diffusion coefficient histogram in Figure 4g. The bins are colored by their probability. (c) 2D histogram of the virus diffusion coefficient versus the track duration. The bins in *x*-direction are identical to, and normalized by, the track duration histogram in Figure 4h. The bins in *y*-direction are identical to, and normalized by, the diffusion coefficient histogram in Figure 4g. The bins are colored by their probability.

Apart from directional bias, receptor density dependence may also be expected in virus diffusion speed and dissociation rate. As visualized in Figure 1, a virus attached to the surface at a low receptor density is expected to bind with few receptor–ligand interactions, which allows it to move more swiftly at the expense of a higher probability of dissociation, whereas the opposite is true at high receptor densities. Figure 6a shows a 2D histogram of the track duration versus receptor density. The binning in this histogram was normalized for the relative probabilities of the receptor densities and track durations. The resulting histogram was colored by an exponential color map to enhance the contrast.

The effect of increasing track duration with receptor density is small but noticeable. The bins with high probability (Figure 6a) are located in an area spanning roughly from the lower left to the middle right of the histogram, and more blue color is found in the top right of the histogram in Figure 6a. Yet, all ranges of track durations are observed at all densities, which indicates that, while there is a (small) correlation of track duration with receptor density, it is far from being the only determining factor. Another effect that the previous section touched upon is that the receptor density may fluctuate locally. As the size of a pixel is significantly larger than what a virus can probe, the receptor densities as felt by the virus will differ slightly from the average receptor density in the pixel.

The receptor density-dependent diffusion constant of viruses is shown in Figure 6b. The binning is a combination of Figure 3e and Figure 4g, with the probabilities normalized to these histograms as well. In the lower half of the figure there is some dependence on receptor density. The top half of the image is mostly empty, except for the low receptor densities.

The trend in Figure 6b is much more clear-cut than in Figure 6a, with the higher diffusion constants clearly at lower receptor densities. This is in line with the literature, where multiple populations of viruses were found, distinguished by their mobility.^7^ The explanation that was given is that these populations differ in the number of interactions with the surface. In the case of our data, the lower movement speeds are not confined to any specific receptor density, which indicates that the slow movement of these viruses is not related to the number of receptors they bind.

Combining the track duration information on Figure 6a and the diffusion information on Figure 6b yields Figure 6c. There is a clear, exponentially decreasing trend of diffusion constant versus track duration, though with a relatively high density of bins at the bottom of the figure. The exponential decrease in Figure 6c confirms and explains the density dependence in Figure 6a,b. If the trends of track duration and diffusion constant in Figure 6a,b are indeed receptor density-dependent and caused by the number of virus–surface interactions, they should be anticorrelated. Figure 6c clearly shows the expected anticorrelation as there is a range of different track durations at low diffusion coefficients and a range of diffusion coefficients at low track duration, while the combination of medium/high track durations and medium/high diffusion coefficients is absent. The trend in Figure 6c is even stronger than that in Figure 6b, which is another indication that the receptor density as derived from the fluorescence in a pixel is not always identical with the local environment of a virus in that pixel area.

In summary, the data in this section shows a clear receptor density dependence for both track duration and diffusion coefficient. The duration of the tracks increases with receptor density, while the diffusion coefficient decreases with receptor density. Furthermore, a strong anticorrelation is observed between track duration and diffusion coefficient, which is further evidence that these are determined by the same underlying parameter: the number of interactions between virus particle and surface.

## Conclusions

Based on the behavior of molecular spiders,^16^ influenza particles bound at a surface with a receptor density gradient were expected to exhibit a preference for directional motion toward higher receptor density. In this work, such a directional bias was not observed. Most likely, the steepness of the gradient was insufficient to show this effect. In a biological setting, continuous gradients of receptor density at a cell surface over macroscale distances are not expected. The glycocalyx is highly heterogeneous, and thus, receptor densities on a cell surface may vary, rather gently, at some locations with more sudden changes at other locations. Receptor densities will also vary between cells, cell types, or cell–mucus boundaries. Influenza may exhibit a directional preference at the steeper boundaries, but that could not be studied using the surface gradients in this study. Since the receptor densities studied here already go beyond the average protein–ligand site densities of a virus,^12^ further gradient design may need to be developed along existing theory.^37^

The surface mobility, however, was found to be significantly dependent on receptor density, which agrees well with the other literature.^7^ The combination of high surface mobility and high dissociation probability of influenza at low receptor densities, and the opposite behavior at higher receptor densities, results in an effective mechanism for finding high-receptor density patches, which plays an important role in the cellular uptake of influenza.

The density-dependent behavior found in this study identifies another connection between the activities of the HA and NA proteins of influenza. HA and NA actions balance mobility and binding, but this balance also shifts as a function of receptor density. This implies that, when interpreting antigenic changes in HA and NA between influenza strains, one should not just consider receptor type and affinity, both absolute and relative between HA/NA, but also receptor density.

## Supporting Information Available

Discussions of materials and methods used and experimental details and figures of fluorescence micrograph of labeled SAv, receptor density, fluorescence micrograph of fluorescent dye-labeled influenza viruses, localization accuracy, histogram of track duration, and reducing the dimensionality of data (PDF)
