# Supplementary material for: Receptor Density-Dependent Motility of Influenza Virus Particles on Surface Gradients
Source: ACS Appl Mater Interfaces. 2023 May 11;15(20):25066–76. doi: 10.1021/acsami.3c05299 (PMC10214370; doi:10.1021/acsami.3c05299)
Supplement: Supplementary file 1 — am3c05299_si_001.pdf [file am3c05299_si_001.pdf]

Supporting Information

Receptor Density-Dependent Motility of Influenza Virus Particles on Surface Gradients

P. H. (Erik) Hamming,[a] Nico J. Overeem,[a] Kevin Diestelhorst[b], Tren Fiers,[a] Malte Tieke,[c] Gaël M. Vos,[d] Geert-Jan P. H. Boons,[d,e,f] Erhard van der Vries,[c,g,h] Stephan Block,*[,b] and Jurriaan Huskens*[,a]

E-mail: stephan.block@fu-berlin.de; j.huskens@utwente.nl

[a] Molecular Nanofabrication Group, MESA+ Institute, Faculty of Science and Technology, University of Twente, P.O. Box 217, 7500 AE Enschede, The Netherlands

[b] Institute of Chemistry and Biochemistry, Freie Universität Berlin, 14195 Berlin, Germany

[c] Division of Virology, Department of Infectious Diseases and Immunology, Faculty of Veterinary Medicine, Utrecht University, 3584 CL Utrecht, The Netherlands

[d] Department of Chemical Biology & Drug Discovery, Utrecht Institute for Pharmaceutical Sciences, Bijvoet Center for Biomolecular Research, Utrecht University, 3584 CG Utrecht, The Netherlands

[e] Complex Carbohydrate Research Center, University of Georgia, 315 Riverbend Rd, Athens, GA 30602, USA

[f] Department of Chemistry, University of Georgia, Athens, GA 30602, USA

[g] Royal GD, Arnsbergstraat 7, 7418 EZ Deventer, The Netherlands

[h] Department of Clinical Chemistry and Haematology, University Medical Center Utrecht, Utrecht University, 3584 CX Utrecht, The Netherlands

Materials and methods

Materials

The biotinylated glycan 2,3-S(LN)$_3$ was synthesized as described before.[1] Influenza A/Puerto Rico/8/34 virus (Mt. Sinai strain) stocks were prepared and labeled as described before.[1] All other starting materials and chemicals were purchased from commercial suppliers and used as received, unless otherwise stated.

Chip fabrication

Flow cells were fabricated according to the procedure described earlier.[2] A bilayer lift-off recipe was used for fabricating Au electrodes on Mempax glass wafers of 0.2 mm thickness (Schott). First, hexamethyldisilazane (HMDS) was spin-coated, after which normal lithography was performed on top with Olin OiR 907-17 photoresist (FujiFilm) to create a bilayer resist stack. Electrode patterns were made by exposing the photoresist through a patterned photomask and developing in Olin OPD 4262 (FujiFilm). The develop step washed away theexposed photoresist, and etching through the LOR 5A layer created an undercut. Then, 5nm Ti and 95 nm Au were deposited via sputtering (T'COathy machine, MESA+ NanoLab, University of Twente). The bilayer resist was then removed by sonication in acetone (20 min) and isopropanol (10 min), serving as a sacrificial layer to leave patterned Au electrodeson Mempax glass. To fabricate the Cr corrals (10 nm thick) in between the Au electrodes, the same procedure was performed a second time, but in this case following alignment with respect to the Au electrodes.

PDMS flow channel

Silicon flow channel masters were produced by standard photolithography steps and deep reactive ion etching. The polydimethylsiloxane (PDMS) flow channels were prepared from a degassed mixture of 10:1 Sylgard 184 elastomer and curing agent (Dow Corning Corp), which

was cast onto the silicon master and cured at 60 °C overnight. The flow channels were cut to size and inlets and outlets were punched using a 1 mm Ø punch (Harris Unicore, Sigma-Aldrich). After bonding to the chip, a flow channel of 6,000 x 500 x 50 µm was prepared with a second channel entering from the side.

PDMS bonding

Chips were rinsed and sonicated extensively with acetone, ethanol and MilliQ water, and dried prior to UV-ozone exposure (UV/Ozone Procleaner plus, Bioforce Nanosciences) for at least 20 min. After UV exposure, the chips were rinsed with ethanol and water, and dried under a stream of nitrogen. Both cut-out PDMS flow channels and cleaned chips were treated with oxygen plasma for 30 s at 40 W (Plasma prep II, SPI supplies) after which they were bonded immediately. The chips were placed on a hot plate for 10 min at 70 °C to increase the binding strength. Tygon Microbore tubing S-54-HL (VWR, 0.25 mm inner Ø and 0.75 mm outer Ø) of 80 cm for the inlets and 40 cm for the outlets was inserted into the PDMS. The assembled flow cell was placed in an oven at 60 °C for 1 h. Leak-free operation was shown for flow rates up to 200 µL/min.

Lipid vesicle preparation

The lipid 1-myristoyl-2-palmitoyl-sn-glycero-3-phosphocholine (MPPC) was stored as a 10 mg/ml stock solution in chloroform at -20° C. 1,2-Dioleoyl-sn-glycero-3-phosphoethanolamine-*N*-(biotinyl) (DOPE–biotin) was stored as a 0.2 mg/ml solution. Aliquots of these stocks with desired lipid molar ratios were mixed in a glass vial, dried under a flow of nitrogen and kept under vacuum for at least 1 h. The resulting film was resuspended by vortexing in MilliQ water at 50 °C to form multilamellar vesicles (MLVs) at 1 mg/ml. The MLV solution was extruded 11 times through a 100 nm polycarbonate membrane (Avanti) at 50 °C. The resulting small unilamellar vesicles (SUVs) were kept at room temperature and used within one week.

SLB formation and functionalization

Before SLB formation, the flow cells were washed with 2% SDS (Sigma-Aldrich), then rinsed with MilliQ, and washed overnight with 2% Hellmanex to activate the glass surface. Flow cells were mounted onto a heating plate using Scotch tape and rinsed with MilliQ and PBS, while heating to 50 °C. Shortly before SUV incubation, the SUV solution was diluted to 0.5 mg/ml with PBS. The diluted SUV solution was passed through the flow cells through the secondary inlet for 30 min (10 µL/min; primary inlet 1 µL/min PBS) to allow adsorption and rupture of the vesicles on the chips. The chips were then washed with MilliQ (100 µL/min secondary inlet, 10 µL/min primary inlet), after which freshly prepared 0.5 mM hydroxymethylferrocene (FcMeOH, Acros) was passed through the device (50 µL/min, bothinlets). A potential difference of 2.0 V was applied over the device for 30 min to induce electrophoresis in the SLBs. Subsequently, the chips were cooled rapidly on a heat exchanger to fix the surface gradient. The flow cells were then rinsed with MilliQ. Bovine serum albumin (Sigma) 50 mg/ml (0.5 ml, 10 µL/min) was passed through the flow cells to forman antifouling layer on the tubing. The flow cells were rinsed with PBS. SAv with Alexa Fluor 488 label (Thermo Fischer) 20 µg/ml was passed through the secondary inlet (10 µL/min, primary inlet 1 µL/min). The flow cells were rinsed with PBS. The SAv-modified SLBs were inspected with fluorescence microscopy. A solution with 500 nM of the glycan 2,3-S(LN)$_3$-biotin was passed through the primary inlet (10 µL/min, and secondary inlet 1 µL/min), after which the flow cells were rinsed with PBS. During all steps, care was taken to ensure that no air bubbles entered the flow cell.

Gradient analysis

The local receptor density in each pixel, $\rho_{R,i}$, may be calculated using

$$\rho_{R,i} = \bar{\rho}_R * \frac{I_i - \bar{I}_{bg}}{\bar{I} - \bar{I}_{bg}} \quad (1)$$

with $\bar{\rho}_R$ the average receptor density based on the percentage of biotin-DOPE in the SLB and the lipid footprint (60 Å^2), I_i the local fluorescence intensity, $\bar{I}_{bg}$ the average fluorescence intensity of the background, and $\bar{I}$ the average intensity of the (full) corrals.

Virus experiments

Flow cells with 0.2 mm glass were used to allow higher magnifications. Gradients were formed from SLBs with 0.5% biotin-DOPE and were functionalized with SAv and biotinylated 2,3-S(LN)$_3$. Virus solution was passed through the microchannel at a flow rate of 5 µl/min. Fluorescence micrographs were acquired on a Nikon Ti-E fluorescence microscope with perfect focus system and Andor Zyla 4.2 sCMOS camera at 40x magnification with oil immersion. Blue excitation (473≤ λ_{ex} ≤491 nm) and green emission (506≤ λ_{em} ≤548 nm) were filtered to image the SAv gradient, and green excitation (542≤ λ_{ex} ≤582 nm) and red emission (602≤ λ_{em} ≤678 nm) were filtered to image the viruses. An Andor Zyla VSC-07418 camera was used to acquire images in 16-bit dynamic range. Virus binding was studied by acquiring fluorescence micrographs at 5-s intervals.

Tracking method

Micrograph image sequences were analyzed using the TrackMate plugin of Fiji.[3,4] Individualvirus spots were detected using a Laplacian of Gaussian filter, which specifically detectscircular blobs close to a specified diameter. In this case a diameter of 1.4 µm was used, asthat is the approximate size of a virus diffraction spot in our setup. The coordinates of eachspot are localized to sub-pixel accuracy using a quadratic fit,[5] which is built into TrackMate.

Tracks were constructed from the list of spots using the "simple LAP tracker" built into TrackMate, based on the work of Jaqaman *et al.*[6] This is a general-purpose particle trackerthat

attempts to link the spots of consecutive frames, followed by a global (*i.e.*, operatingover all frames at the same time) optimization to link the track segments. The maximumdistance between spots or track segments to be linked was set at 1.5 µm, which is slightlylarger than the size of a virus diffraction spot. The maximum time between track segmentsto be linked was set to 10 frames, which is needed as sometimes virus flowing in solutionobstructs the view of the surface. With these conditions, over 95% of the links between spotsis between consecutive frames and 95% of the links between spots is over distances less than 0.37 µm.

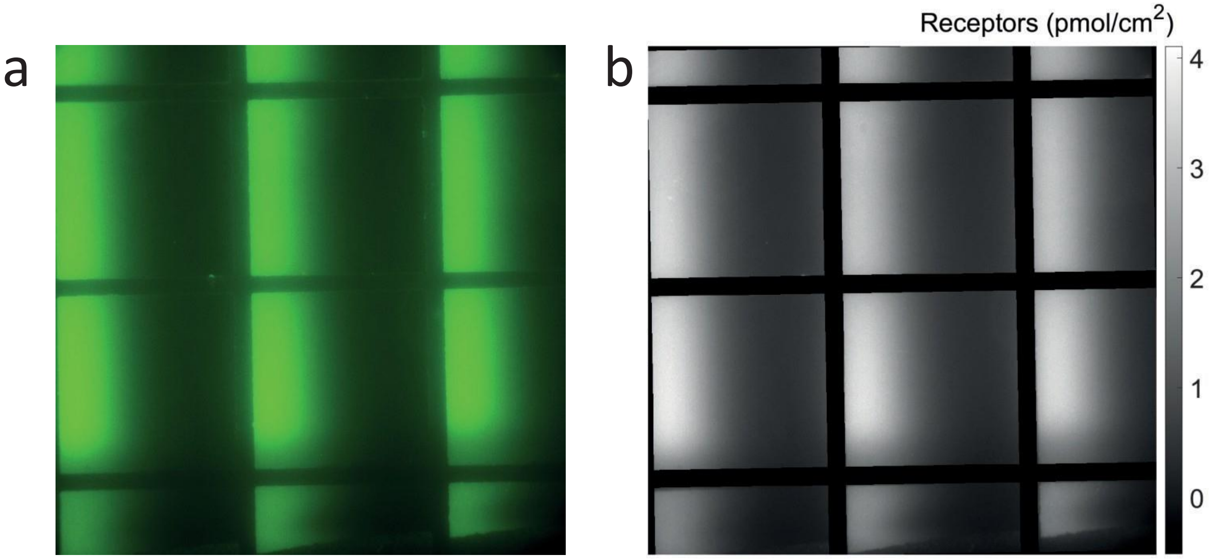

Figure S1. Receptor gradients. (a) Fluorescence micrograph of labeled SAv, showing the receptor density gradient on an SLB containing 0.5% biotin-DOPE. Square corrals are 100x 100 µm. (b) Receptor density as calculated from the data in (a), based on Equation 1. The grayscale value of each pixel represents the receptor density. The normalization was calculated based on the complete corrals and extended to the visible area.

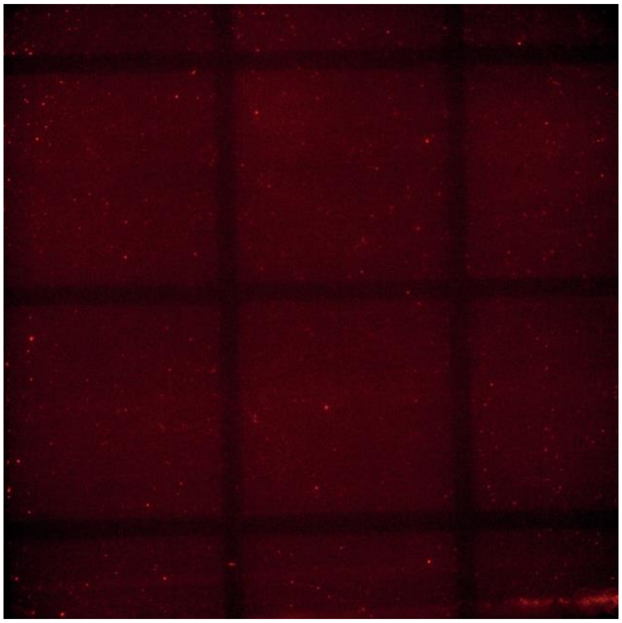

Figure S2. Fluorescence micrograph of fluorescent dye-labeled influenza viruses. The brighter spots can easily be identified, but the darker spots are difficult to discern.

Localization accuracy

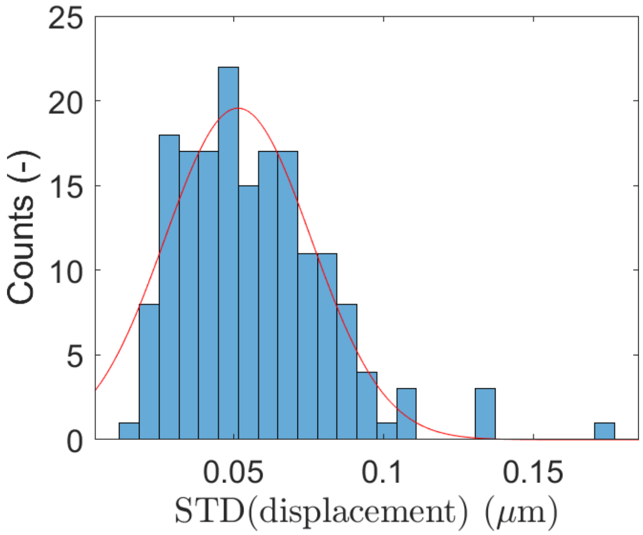

Figure S3. The localization accuracy was determined by calculating the standard deviation of the displacement for tracks longer than 250 frames (out of 252 total): histogram of these standard deviations with a Gaussian fitted on it. The center of the Gaussian is at 0.052 µm, which indicates that the localization accuracy of our method is 52 nm.

Distributions of virus track durations

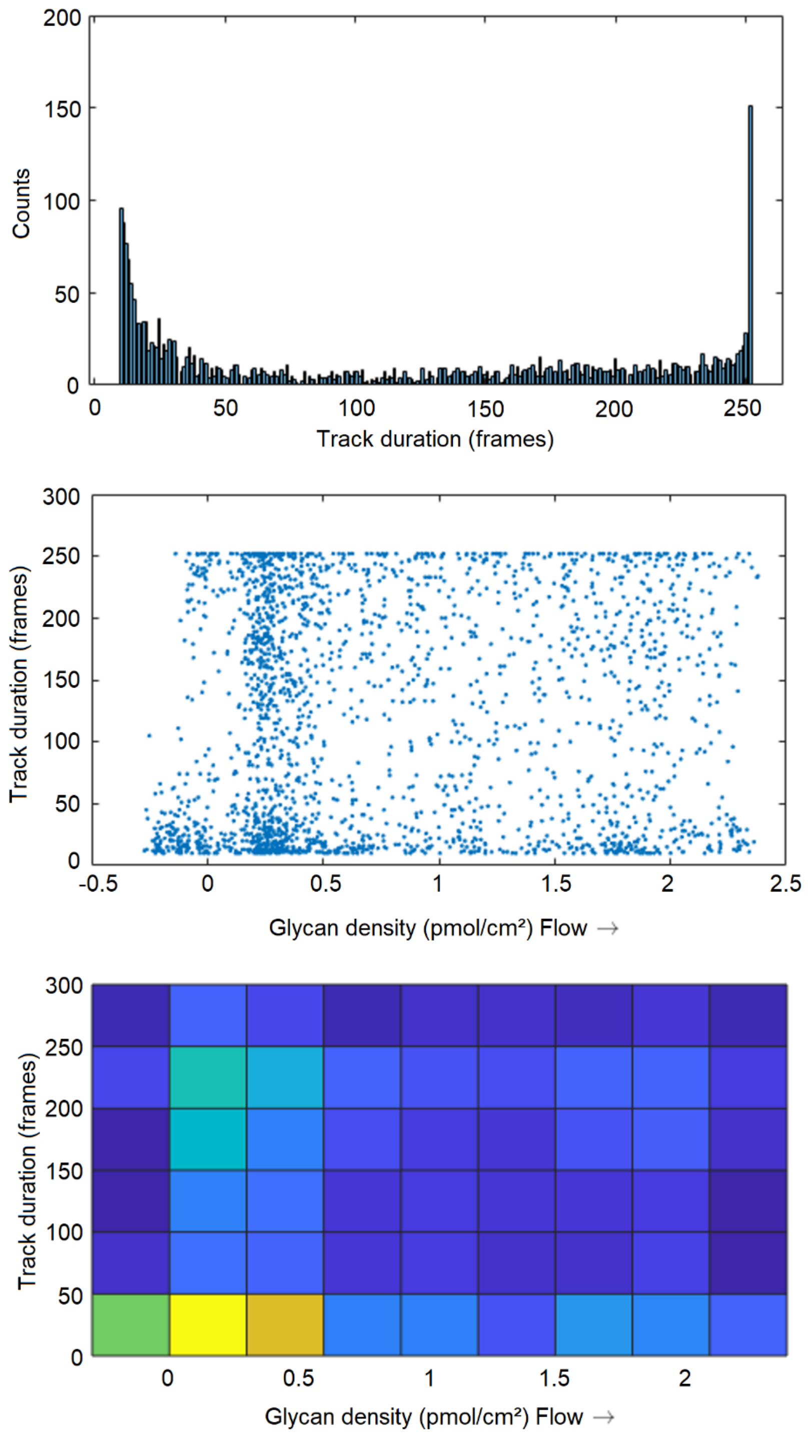

Figure S4. Histogram of track duration for tracks of at least 10 frames (top, same as Figure 3c). Center and bottom give different views of the track duration as a function of glycan density. The rather homogeneous distribution of both shorter (<50 frames) and longer (>50 frames) (compare also to Figure 4e/h) confirms that both mobile and immobile fractions are distributed independently of glycan density.

Reducing dimensionality

Reducing the dimensionality of the data makes quantitative analysis computationally easier. This representation of the data makes it easier to separate random motion (average slope zero) from any biased movement (average slope non-zero) and identify transient effects (non-linear slope). As the starting time of each track is not of interest, they are synchronized to start at time=0.

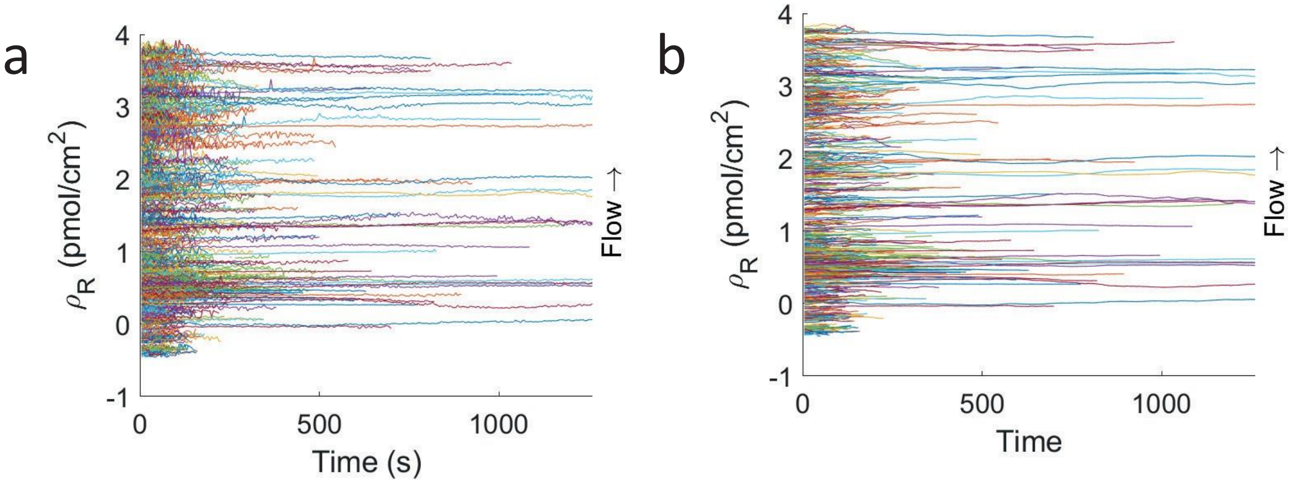

Figure S5. Reducing the dimensionality of data. (a) Tracks of mobile viruses shown as receptor density over time, instead of (x,y) over time, and synchronized to start at t=0. (b) Same data as (a), but filtered with a +/- 4 frames moving mean to bring out longer timescale features of the tracks.

References

(1) Overeem, N. J.; Hamming, P. H. E.; Grant, O. C.; Di Iorio, D.; Tieke, M.; Bertolino, M. C.; Li, Z.; Vos, G.; de Vries, R. P.; Woods, R. J.; Tito, N. B.; Boons, G.-J. P. H.; van der Vries, E.; Huskens, J. Hierarchical Multivalent Effects Control Influenza Host Specificity. *ACS Central Sci.* **2020**, *6*, 2311–2318.

(2) Overeem, N. J.; Hamming, P. H.; Huskens, J. Time-Dependent Binding of Molecules and Nanoparticles at Receptor-Modified Supported Lipid Bilayer Gradients in a MicrofluidicDevice. *ChemistrySelect* **2020**, *5*, 9799–9805.

(3) Tinevez, J.-Y.; Perry, N.; Schindelin, J.; Hoopes, G. M.; Reynolds, G. D.; Laplantine, E.; Bednarek, S. Y.; Shorte, S. L.; Eliceiri, K. W. TrackMate: An Open and Extensible Platform for Single-Particle Tracking. *Methods* **2017**, *115*, 80–90.

(4) Ershov, D.; Phan, M.-S.; Pylvänäinen, J. W.; Rigaud, S. U.; Le Blanc, L.; Charles- Orszag, A.; Conway, J. R. W.; Laine, R. F.; Roy, N. H.; Bonazzi, D.; Duménil, G.; Jacquemet, G.; Tinevez, J.-Y. Bringing TrackMate into the Era of Machine-Learning and Deep-Learning. *bioRxiv* **2021**, 2021.09.03.458852.

(5) Lowe, D. G. Object Recognition from Local Scale-Invariant Features. Proceedings of the Seventh IEEE International Conference on Computer Vision. 1999; pp 1150–1157 vol.2.

(6) Jaqaman, K.; Loerke, D.; Mettlen, M.; Kuwata, H.; Grinstein, S.; Schmid, S. L.; Danuser, G. Robust Single-Particle Tracking in Live-Cell Time-Lapse Sequences. *Nature Methods* **2008**, *5*, 695–702.
